# Nurses’ and teachers’ perceived barriers and facilitators to the uptake of the Human Papilloma Virus (HPV) vaccination program in Iquitos, Peru: A qualitative study

**DOI:** 10.1371/journal.pone.0255218

**Published:** 2021-07-29

**Authors:** Anna Clavé Llavall, Gilles de Wildt, Graciela Meza, Jasmine Tattsbridge, Laura Jones

**Affiliations:** 1 University of Birmingham Medical School, University of Birmingham, Birmingham, United Kingdom; 2 Institute of Clinical Sciences, University of Birmingham, Birmingham, United Kingdom; 3 Facultad de Medicina Humana, Universidad Nacional de la Amazonia Peruana, Iquitos, Perú; 4 Institute of Applied Health Research, University of Birmingham, Birmingham, United Kingdom; George Washington University, UNITED STATES

## Abstract

Globally, over 300,000 women die of cervical cancer annually. Given that human papillomavirus vaccines are highly effective in the primary prevention of cervical cancer, it is important to explore the barriers and facilitators to vaccination uptake in areas where the burden of disease remains high. This study, informed by the socio-ecological model, aimed to qualitatively explore vaccination uptake via in-depth interviews with eleven nurses and ten teachers involved in vaccine delivery in Iquitos, Peru. The results highlighted that vaccine uptake was influenced by multiple factors including individuals’ knowledge and attitudes, community beliefs, geography, and policy level variables. Findings suggested that professionals were informed and supportive of the HPV vaccination program but perceived that parents were uninformed about the vaccine. There is a need for community education programs, for a revision of the process of obtaining parental consent, for improved communication between professionals and for involvement of grassroots staff in policy making.

## Introduction

Human papillomavirus (HPV) is the most common sexually transmitted infection worldwide [1]. The global prevalence is estimated to be 10.4% within the female population [1]. Over 100 genotypes of HPV have been identified, thirteen of which are classed as high-risk [2]. Persistent infection with these types of HPV can cause cervical cancer as well as other anogenital and oropharyngeal neoplasms [2].

In 2020, 604,127 women were diagnosed with and 341,831 (56.6%) died of cervical cancer globally [3]. Low- and middle-income countries (LMICs) suffer the highest disease burden. The disease is not just more common in LMICs (18.8 cases per 100,000 versus 11.3 cases in high income countries) but mortality is also higher, with LMICs suffering 90% of the total number of cervical cancer-related deaths [3]. It is the leading cause of death due to malignancy among 20-40-year-old women in Latin America [4]. In Peru, the cervical cancer age-standardized incidence rate is 22.2 per 100,000 (world average 13.3) [3]. This translates to 4270 new cases and 2288 deaths annually [3].

HPV genotypes 16 and 18 contribute to 70% of cervical cancer cases [5]. Two vaccines protecting against these subtypes are available: Gardasil and Cervarix [6]. They are most effective if delivered before sexual initiation i.e. before initial infection with HPV [6]. It has been estimated that if the vaccine was given to 70% of twelve-year-old girls in Latin America and the Caribbean over ten years, half a million deaths could be prevented [7].

Peru was the first country in South America to introduce a national HPV vaccination program in 2011 [8]. Currently two doses of the vaccine are given to 9-13-year-old girls [9]. This replaced the previous recommendations of giving three doses to girls in school grade 5 (10–11 years of age) [6,9]. Local schools and health centers work together to deliver the vaccination. Every primary health center is responsible for a certain number of neighboring schools. Healthcare professionals from the health center approach the educational centers to agree on specific dates for the vaccination to be administered to the appropriate student population on school grounds [10]. However, coverage rates remain low and vary between years. The average coverage was 34.9% in 2011, falling to 6.4% in 2016 [11].

There are also geographical variations. Although the cervical cancer incidence rate has fallen in Lima, it remains high in other regions [12]. The northeast region of Loreto suffers the highest adjusted cervical cancer mortality rate in the country [13]. These differences arise due to various factors which include poverty, disparities in education and difficulty accessing healthcare [12]. In Loreto, the HPV vaccination rates were 19.21% in 2011 and had fallen to 0.6% in 2016 [11]. In addition, Loreto has high rates of risk factors associated with HPV infection [14]. For example, the 2017 Demographic and Health Survey (ENDES) found that women in the region have a lower median age at first sexual intercourse (16.6 years versus the national average of 18.5 years) and a fertility rate of 3.7 (national average 2.4) [15]. Additionally, only 46.1% of 15-59-year olds associate HPV with cervical cancer [16].

Research in Peru has focused on exploring parents’ and women’s attitudes towards HPV vaccination [17,18]. Such studies found that 25-65-year-old women have little knowledge about the association between HPV and cervical cancer but show high acceptability of the vaccine [17]. When investigating the thoughts of the girls’ parents, fear of side effects and a perceived lack of information surrounding the vaccine were found to be barriers to acceptability [18]. Contrastingly, numerous studies have highlighted the importance of healthcare provider recommendation as a facilitator to HPV vaccination uptake [19–24].

To the best of the authors’ knowledge, there are no published studies that have explored the attitudes and experiences of professionals involved in the delivery of the HPV vaccination program in Peru. Additionally, most of the research surrounding the HPV vaccine has been conducted in high-income countries. Their findings may therefore not be transferable to LMICs, which suffer the highest burden of disease, because of differences in healthcare systems and culture [25,26]. It is also necessary to understand why there is variation in vaccine uptake between regions. As a result, it is important to explore attitudes and perceptions about the vaccine among local professionals. This qualitative study aims to explore and understand: (1) local nurses’ and teachers’ perceived barriers and facilitators to the implementation of the HPV vaccination program, (2) knowledge and attitudes towards HPV, (3) recommendations on strategies that could be used to increase vaccination rates, and (4) experience regarding professionals’ training and the public’s education about the vaccine.

## Methods

### Setting

The study was conducted in Iquitos, the capital city of the Amazonian region of Loreto, in North-East Peru. It has a population of 437,376 (2015) and can only be accessed by boat or plane [27]. The region’s population is sparsely distributed with a population density of 3.2 people per km^2^ (national average 24.8 people per km^2^) [28] and 35.2% of the population lives below the poverty line (national average 22.7%) [29]. Locals largely self-identify as mestizo (mixed-race) [30].

### Study design and guiding framework

**Fig 1:**
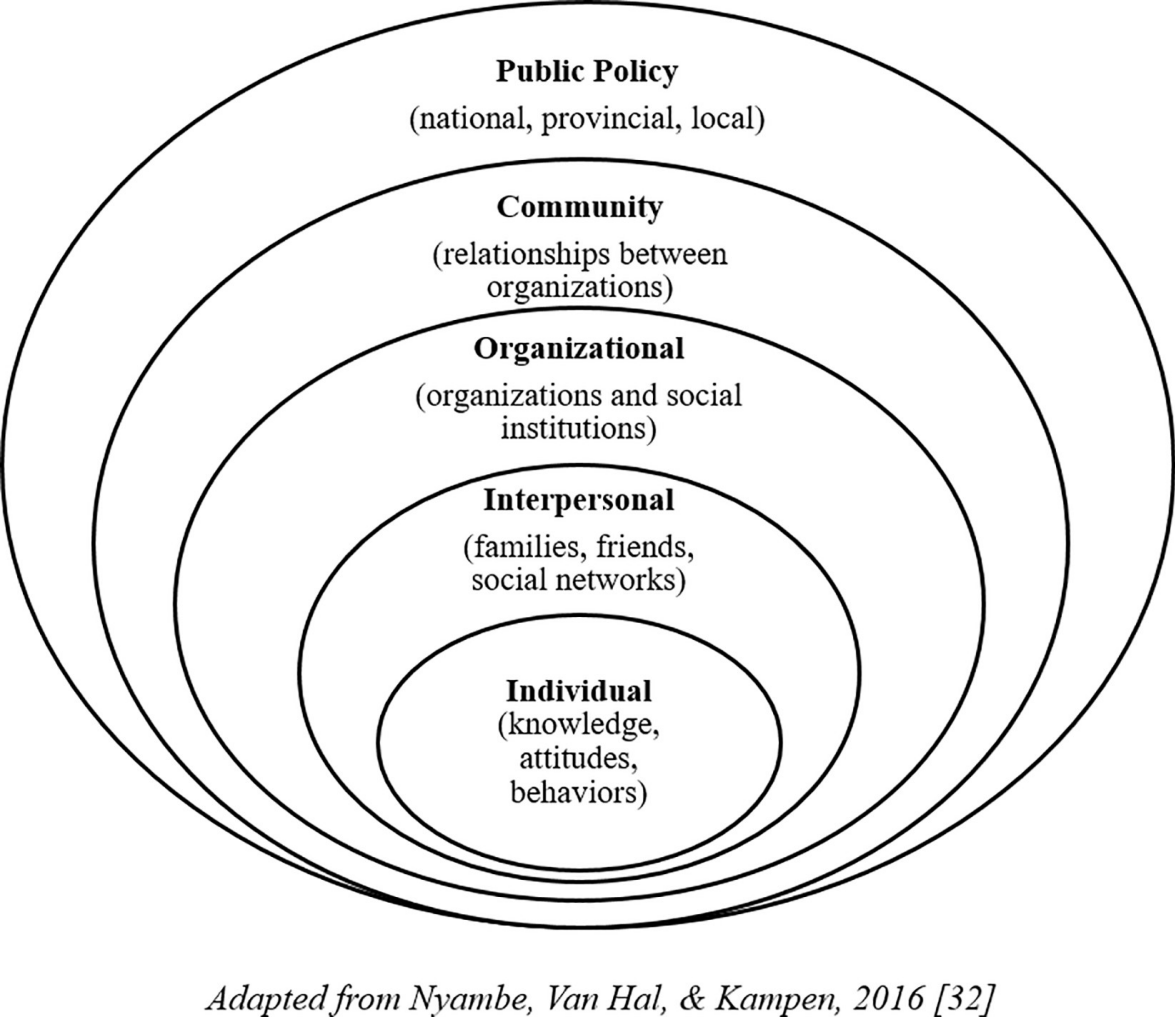
Five-level socio-ecological model.

The qualitative design allowed for the development of in-depth understanding of the participants’ knowledge, attitudes and perceptions [31]. The five-level socio-ecological model (SEM) was used as the theoretical framework that informed the study (Fig 1) [32]. The SEM model recognizes the intertwined relationship between individuals and their environment and that public health problems, such as low vaccine uptake, are complex and affected by multiple levels of influence, and can therefore not be investigated using a single-level analysis [32,33]. The model informed data collection, analysis, and interpretation.

### Sampling

Nurses and teachers involved in the delivery of the HPV vaccine program were initially purposively sampled [34]. Diversity of both teachers and nurses in terms of participant age, length of time in program, location of work (rural versus more urban environments) was sought. Exponential non-discriminative snowball sampling was then used to facilitate further recruitment [35].

### Recruitment

Recruitment took place in the last two weeks of January and throughout February 2019. The principal investigator (A1), and the local supervisor (A3) approached the health centers and schools to explain the study to the nurse in charge or relevant school director. Once they were aware of the study and its implications, they identified eligible participants. Participants were eligible to participate if they met the following criteria: a nurse involved in the HPV vaccine delivery process or a primary school teacher who worked with the HPV vaccination program; Peruvian citizen resident of Loreto; over 18 years of age; speaking either Spanish or English fluently; willing to participate and able to give informed consent. A1 then approached potential participants, checked their eligibility, explained the study, provided a participant information sheet, and answered any queries. If they agreed to participate, written informed consent was obtained and a suitable time for the interview was identified. It was also emphasized that participation was voluntary and that their information would remain confidential.

### Data collection

This qualitative study involved semi-structured interviews with nurses and teachers. Individual interviews were chosen as the main form of data collection because of the study’s aim to gain insight into individuals’ perceptions, which are more likely to be elicited in one-to-one interviews rather than in a group setting [36]. Interviews were conducted in a private room at the participants’ workplace. The semi-structured, face-to-face interviews followed a topic guide (S1 File) developed using existing literature [37,38] and informed by the five-level SEM. A1 conducted the interviews in Spanish. All interviews were audio recorded and then transcribed verbatim in Spanish and translated into English by A1. The transcripts were anonymized. Field notes and initial observations were noted to maximize trustworthiness and researcher reflexivity. Before starting each interview, participant socio-demographic information was collected using a demographic questionnaire.

### Data analysis

Data analysis was informed by the Braun and Clarke’s 6-step thematic approach [39]. Recruitment was conducted in parallel to analysis and continued until the research team deemed that the data were sufficiently rich to address the study aims [36].

Data immersion was achieved through repeated reading of the transcripts and by subsequently translating them into English. NVivo 12 software was used to support the analysis process. The SEM was initially applied to the data deductively, and then inductive coding was developed within each of the five SEM levels. A thematic mind map was developed to assist with the process of reviewing the initial themes [39]. Analytical triangulation was performed to increase reliability [40] by having a second researcher (A4) code and validate a sample of two data-rich, anonymized transcripts. Any discrepancies between the two researchers were discussed and agreed on. Further analysis and interpretation of data was supported by the wider research team (A2, A3, A5). Descriptive analysis of the demographic questionnaire data was undertaken using IBM SPSS Statistics 24 software.

### Ethics statement

Ethical approval was obtained from the University of Birmingham Internal Ethics Review Committee and the Institutional Research Ethics Committee at the Department of Health Loreto. Informed written consent was obtained from all participants prior to data collection.

## Results

### Participants

Twenty-one interviews (average length 30 min, range 10–56 min) were conducted with eleven nurses working in two primary health centers, and ten teachers from four public primary schools in the area. All professionals were or had been involved in the delivery of the HPV vaccine program. Four teachers were purposively sampled with the remaining six snowballed via their colleagues. For nurses, seven were purposively sampled and four were snowballed via their colleagues. All the participants self-identified as “mestizo” (mixed-race) and most were female (85.7%). To preserve anonymity, a summary of the participant demographics is presented in Table 1.

**Table 1:** Summary of participant demographics.

Characteristic Number (%)	Nurses (n = 11)	Teachers (n = 10)	Total sample (n = 21)
Women	11 (100)	7 (70)	18 (85.7)
Individuals who received specific training on HPV vaccine			
Yes	10 (91)	7 (70)	17 (81)
No	1 (9)	3 (30)	4 (19)
**Characteristic Mean (range)**			
Age in years	38.5 (30–48)	52.8 (41–61)	45.3 (30–61)
Years licensed	11.2 (3–19)	27.2 (8–38)	18.8 (3–38)
Years in current job	5.7 (1–17)	14.9 (2–27)	10.1 (1–27)
Years of experience working with HPV vaccination program	4.1 (1–7)	2.6 (1–8)	3.4 (1–8)

### Findings

Five core themes were interpreted inductively: (1) knowledge, (2) acceptance, (3) accessibility, (4) communication and (5) resources. All core themes were relevant to more than one level of the socio-ecological model and therefore the results are presented by inductive theme rather than SEM level. Table 2 summarizes the findings and links the subthemes to the five SEM levels. Interpreted subthemes included: well informed participants, perceived limited awareness of parents, high vaccine acceptability, and girls’ fear of the vaccine (individual level), issues with parental consent, school attendance, and the influence of familial customs and religion (interpersonal level), staff shortage, occupational risks, and problems surrounding the program’s funding (organizational level), beliefs and social norms, geography and climate, the influence of media and the internet, and communication between education and health (community level), and strategies such as running awareness raising campaigns, increasing the program’s target group, improving the recording system, making the vaccine compulsory, and improving decision-making (policy level). An interpretation of each of the themes is presented below with illustrative quotations.

**Table 2:** Summary of interpreted core themes and subthemes modelled against the five levels of the socio-ecological model.

CORE THEMES	FIVE LEVELS OF THE SOCIO-ECOLOGICAL MODEL				
	Individual	Interpersonal	Organizational	Community	Public policy
**Knowledge**	Basic knowledge of participants Desire for more information Perceived limited awareness of parents			Media and internet as sources of information	Awareness raising campaigns
**Acceptance**	High parental acceptance Girls’ fear High acceptability among teachers/nurses	Parental Consent Influence of familial customs and religion		Beliefs and social norms	Making vaccination compulsory
**Accessibility**		School attendance (migration, dropping out)		Geography and climate	House to house campaigns, increasing target group
**Communication**				Health-education communication	Centralized recording system
**Resources**			Staff/material shortage ccupational risks Funding		Corruption Decision-making processes

#### 1) Knowledge

As outlined in Table 2, knowledge aligns with several levels of the SEM. At an individual level, there was widespread basic knowledge on HPV and the HPV vaccine amongst all participants. Most knew about the virus and its relationship with cervical cancer and talked about the vaccine as a preventative measure: *“We know it’s [HPV vaccine] for prevention” (Nurse)*. Many also correctly identified that the virus is sexually transmitted, linked its pathogenic effects to women and acknowledged that men are carriers. Three nurses were able to give more specific details such as high-risk genotypes or vaccine side-effects: *“Genotypes 16*, *18 are the ones that give most cancer”*.

However, all but one participant highlighted the need for more staff training and a desire for more information on the topic. They thought it would be beneficial to get further information to be able to run educational campaigns with parents and girls: *“Many workers*, *even though they are trained (…) forget or don’t know” (Nurse)*. Many agreed they would have liked more specific knowledge on topics such as vaccine statistics, effectiveness, and side-effects.

Two teachers specifically thought that having more information would facilitate their communication with parents. They mentioned episodes where parents confronted them after their child suffered mild side effects associated with the vaccine or instances where parents asked them vaccine-related questions which they were unable to answer appropriately: *“If we knew*, *maybe we could defend ourselves with the parents”*.

Nurses and teachers also perceived a lack of parental knowledge as an important barrier to vaccination. They felt parents were unaware and therefore unable to make an informed decision relating to their child receiving the vaccine. They highlighted the importance of providing parents with information: *“If they lack education*, *sometimes they don’t*, *don’t*, *no*, *no*, *they don’t accept their child getting vaccinated” (Teacher)*.

At a community level, participants also mentioned the importance of media, the internet, and new technologies as platforms to deliver information and HPV related education to the public. They put forward that television and social media, with Facebook being repeatedly mentioned, were good sources of information and a way of reaching lots of people: *“The media has a lot of influence” (Teacher)*. Some admitted to personally using social media to get more information on the topic.

A large proportion of the sample highlighted the need to run more informative sessions at a national policy level. However, there was no consensus on the best strategy to address this. Although many mentioned running informative meetings in schools, others highlighted that many parents did not attend such meetings because of work and family commitments: *“They don’t participate [in school based informative events] much*. *We have that*, *a little absenteeism” (Teacher)*. Two participants thought that handing out leaflets with information about the vaccine could be a good option; however, this was viewed negatively by other interviewees: *“They don’t care*. *They see a piece of paper and they don’t read it*. *They throw it away” (Nurse)*.

There was some consensus that the day of school registration was a good time to deliver information. It was considered a good opportunity because all parents needed to physically attend the school to enroll their children: *“During enrolment (…) all the parents come; at the time of registration they are in masses*. *That’s where we should inform them” (Teacher)*.

#### 2) Acceptance

Most participants thought parental attitudes and beliefs towards the vaccine were positive but identified that there was room for improvement: *“Maybe not 100%*, *but we have had good numbers [of parental acceptance]” (Nurse)*. They believed the education was the best way to increase acceptance rates: *“If you don’t inform people*, *you definitely won’t be able to get there” (Teacher)*. Nurses and teachers reported that parents that rejected the vaccine often did so because of fear of serious side-effects such as paralysis and infertility.

Secondly, participants reported that the girls’ fear of the vaccine was also a barrier to vaccination acceptance. For example, it led to girls missing school if they knew the day vaccination was going to take place, them crying or running away: *“Many times due to fear (…) they don’t come*, *they stay at home” (Teacher)*.

All participants thought there was high acceptance of the vaccine among both nurses and teachers facilitating the running of the program. They thought the vaccine was very important for the nation’s health and spoke positively about the school-based approach. However, two participants highlighted disadvantages of such an approach. One mentioned a teacher who refused to allow the nurses to vaccinate her pupils because of her religious beliefs: *“She [the teacher] did not allow it [the delivery of the HPV vaccination]*, *did not allow it*. *And nobody got vaccinated (…) no child was vaccinated that year” (Nurse)*. A second participant also pointed out this issue, stating that private schools, especially those with a religious association sometimes prevented nurses entering the classrooms.

At an interpersonal level, participants also mentioned the influence of familial customs and religion as an important barrier. This was especially associated with older relatives and parents with lower levels of education: *“In our region there are certain beliefs that are still very present” (Teacher)*. An example of such beliefs was described by a participant. A close family member of hers was diagnosed with cervical cancer. Her family blamed the disease on witchcraft and instead of taking her to her oncology appointments, took her to see a shaman healer. Unfortunately, she passed away.

Furthermore, parental consent was identified as a major factor that can hinder the successful delivery of the program. Currently, nurses provide teachers with consent forms which they then distribute to their pupils for parents to sign prior to the vaccination day. Participants mentioned that it made the vaccination process difficult for various reasons. These included the consent form being lost and the girls signing the form themselves or refusing to give it to the parents: *“We’ve also had difficulty with that [parental consent]*. *Because the girls would bring it and not show it to the parent*. *Or signed it themselves” (Nurse)*. Since the vaccination cannot be given without written proof of parental consent, this was a major barrier to achieving high vaccination coverage.

Finally, looking at a community level, acceptance was a problem among some indigenous groups. There are twenty-nine ethnic communities in the Peruvian amazon [41], many of which have their own culture and language. Participants specifically mentioned the Achuar, the Bora and the Yagua groups. Nurses that had worked with some of these groups mentioned the problems they struggled with: *“They [indigenous people] run away and go to the jungle (…) for them the vaccine is like bringing them the disease”*. This made acceptance of the vaccine and communication with such groups difficult. Two nurses highlighted the importance of speaking to the “Apu”, the tribe ruler, who can act as a gatekeeper. They reported that convincing the tribe leader gave you access to the rest of the community.

A proposed strategy viewed positively by many participants was to make vaccination compulsory or introduce opt-out consent. Their argument was largely linked to the fact that the vaccine was for the welfare of the child. Participants also thought that it would be a way to *“make the parent assume his/her responsibility” (Teacher)*. Some mentioned that it was already compulsory in other countries and compared it to the local anemia screening program, whereby children require a hemoglobin test to enroll in primary school.

#### 3) Accessibility

Although all participants thought that delivering the vaccine in a school setting was a good strategy, various problems associated with accessibility were identified. Firstly, at an interpersonal level, school attendance can limit a girl’s access to the vaccine. Numerous participants explained that migration and dropping out of school due to familial situations were common: *“She is taking care of her little brother and grandparents” (Teacher)*. This affects vaccine access since the girls can only obtain it in a school setting. It also made it harder to complete the dosing schedule (two vaccines, 6 months apart) because finding these girls was quite complicated: *“People migrate a lot*. *Today they’re here*, *tomorrow you don’t know where they are” (Nurse)*. Reasons for migration included moving to the city in search of better work opportunities or due to weather factors, since some rural areas by the riverbed get flooded during the winter months. In contrast, one participant explained a difference between rural and urban areas, observing that migration was not common in the city: *“You hardly see this [migration] in the city” (Nurse)*.

In addition, the region’s geography and climate can also increase the difficulty associated with reaching all girls who are eligible for the vaccination (SEM community level). Various characteristics that hindered accessibility to more remote areas were mentioned by nurses including: “*part of our jurisdiction is flooded” (Nurse)*, *“the route is fluvial” (Nurse)*, *“we have swamps” (Nurse)*, *“our region is so immense” (Teacher)*. All these factors made reaching such communities difficult, time-consuming, dangerous, or even impossible in some circumstances.

At a public policy level, a few participants mentioned house to house campaigns as a strategy to maximize the number of girls that are successfully vaccinated: *“You need to look house to house*. *This*, *that’s the advantage*, *that you find them” (Nurse)*. This is currently done as part of many of the early childhood vaccination programs. Some nurses reported that if they found an unvaccinated girl in the correct age range for the HPV vaccination when running such programs, they asked her to come to the health center to get vaccinated or where possible, tried to bring the vaccine to her.

Some participants mentioned a need to increase the program’s target group, especially for it to include boys: *“Government should buy some for men and women” (Nurse)*. This was thought to be important as they identified men as being the ones that carried the disease. In addition, some asked for the program to also include older women who were not vaccinated in their adolescent years and test negative for HPV infection during cervical cancer screening.

#### 4) Communication

Various issues surrounding communication were interpreted within the data. Firstly, both nurses and teachers were happy with the way the two parties collaborated and defended their good relationship: *“Just as they [teachers] support us*, *we [nurses] are here to support” (Nurse)*.

However, two teachers reported they were unaware about the date of vaccination and believed that hindered the program’s success because they were unable to prepare for their arrival: *“They came all of a sudden (…) there was no time to inform the parents” (Teacher)*. As this teacher explained, not being told what day vaccination was going to take place, further complicated communication between the school and the parents. The second teacher who highlighted this lack of communication stressed the fact that she was unable to tell the girls that vaccination was going to take place to ensure that the girls would attend school and bring the signed consent form: *“Sometimes they catch us off-guard*. *They don’t tell us (…) we cannot speak to the girls” (Teacher)*. This contrasted with other participants’ views regarding girls not attending school due to fear of the vaccine. If they were notified that vaccination was going to take place, some chose not to attend class that day.

A third participant, in this case a nurse with extensive experience working with the program, argued that the way professionals communicated with each other had changed drastically in the last years. She explained that previously, meetings were held regularly between the relevant professionals. Instant messaging via WhatsApp was now the main method of communication. She identified various problems with this, for example that it wasn’t a legally-binding and official tool, and that people did not get to know each other face-to-face and did not feel as personally responsible for the program’s success: *“All information is through WhatsApp*. *We don’t know each other” (Nurse)*.

Aligned with public policy level, participants mentioned the need for a widely accessible and user-friendly centralized student database. Currently, although an online system exists, participants stated that they did not have access to it. To find girls who might have migrated and changed school, many relied on calling or messaging colleagues. However, follow-up in such cases remained hard because often the girls did not give a complete address. When asked about the potential advantage of having a centralized system, participants responded positively: *“It would be useful” (Nurse)*.

The lack of good infrastructure and internet connectivity remains a problem in the region. It makes data logging and sharing information complicated: *“Information is often not available in time*. *The internet is a huge limitation (…) My internet is very slow (…) for each girl I can take up to 15 or 20 minutes (…) it was often exasperating” (Nurse)*.

#### 5) Resources

The limited availability of resources was a common issue reported mostly by nurses, this links in with the SEM’s organizational level factors. Participants complained of staff shortage and requested additional workers. Three specifically mentioned that during vaccination campaigns, when the health centers’ nurses went out to vaccinate in schools, routine consultations were overstretched. In addition, nurses felt they needed to be supported better, especially when travelling to more remote locations. They reported having to use their own money to reach such areas: *“We have to spend our own money (…) because it is our responsibility” (Nurse)*. Another explained that the nurses were expected to reach 100% coverage rates but were not provided with the necessary means. Going out to communities often required numerous resources such as ice to store vaccines and a boat with an engine and a skipper. These were sometimes not easily available: *“Only Jesus Christ walked on water*. *If I walked on water*, *I wouldn’t ask you for the money” (Nurse)*.

Importantly, the lack of economic means did not just put a financial strain on nurses but also put them at physical risk. Four nurses spoke about the dangers of swamps, one suffered a motorbike accident during a campaign and numerous other health-related risks which they were exposed to were mentioned: *“If the snake bites us*, *we die*. *Because it happens*. *It happens*. *Poisonous spiders*, *poisonous vipers” (Nurse)*. They also complained about the lack of a personal health insurance during those campaigns.

Furthermore, three participants spoke about corruption. They described the misuse of existing budgets: *“Instead of serving*, *they [politicians] serve themselves” (Nurse)*. Importantly, they felt powerless and defenseless when trying to denounce it: *“You report it*, *you follow the procedure*, *and nothing” (Nurse)*.

A minority also mentioned a lack of materials as a barrier to vaccination. Specifically, one reported a shortage of vaccinations and two participants reported missing vaccination cards, which are given as proof of vaccine to parents. This further complicated follow-up and completing the dose schedule. When that happened, nurses gave out a prescription instead, saying the girl had been vaccinated. However, nurses acknowledged that was not the correct thing to do and that the piece of paper was often lost.

At a public policy level, a proposed strategy was having relevant professionals involved in the decision-making process. Some nurses felt their opinion was not valued and that the people in power were not aware of the region’s reality and needs: *“One thing is to rule from a desk and the other thing is to come and get to know the reality (…) see reality so you can make all the decisions” (Nurse)*.

## Discussion

### Main findings

This study provides important insight into nurses’ and teachers’ perspectives on the barriers and facilitators to successful implementation of the HPV vaccination program in Iquitos. To the best of the authors’ knowledge this had not been explored previously. Barriers to vaccination were identified at multiple levels of the SEM. The study found that participants were generally well informed about the vaccine. However, they also highlighted that they wanted more training and in-depth information. It is not clear how to best deliver this additional training. A recently published Japanese paper found that delivering a lecture to teachers increased their intention to recommend the HPV vaccine [42]. More novel approaches are also being explored. For example, the online course developed by the Catalan Institute of Oncology (ICO) and the Colombian Cancer Institute [43]. Although its efficacy has not been proven yet, it has had a good uptake with nearly 5000 professionals [43] registering to the course. Ensuring appropriate knowledge and high vaccine acceptability amongst professionals is key, since provider recommendation has been repeatedly identified as an important factor in HPV vaccine uptake [19,20,22]. In addition, our participants perceived high acceptability of the vaccine amongst parents. This was found in previous studies both within Peru [18] and in numerous LMICs, such as the Brazilian Amazon or Indonesia [44].

However, many participants thought parents were uninformed about HPV and the vaccine. This was considered a key barrier to vaccination uptake. Importantly fear of side-effects was the most mentioned concern. Such findings were also reported in a study with Peruvian parents [18]. This lack of awareness needs to be urgently addressed as parents with knowledge about the vaccine show higher acceptance rates, highlighting a need for better educational campaigns [44,45]. Furthermore, multiple qualitative studies, one of them conducted in Peru, report that parents themselves desire more information on the topic to aid decision making, highlighting the need for improved educational and information campaigns [18,23,46]. How to best provide the information needs to be further explored. Many participants suggested organizing parent information evenings. However, issues regarding parental attendance to such events were also noted. Finally, a couple participants expressed negative opinions regarding the provision of information leaflets, explaining that parents were unlikely to read them. Another potential barrier to the effectiveness of written information is the 6.4% illiteracy rate (in people aged 15 and above) in Loreto, reported by national government statistics [47].

As mentioned previously, our participants also reported obtaining written consent as a barrier to vaccination. This was also found to be problematic in other settings [48]. In addition, a report comparing different HPV vaccine delivery strategies in LMIC found that programs with opt-out consent were more likely to achieve high coverage rates [48]. This was also reported by the WHO [49]. A recent qualitative study found that parents in Virginia, the first American state to introduce opt-out consent, expressed concerns regarding health policy makers’ transparency and highlighted that deciding whether one decides to give the HPV vaccine should be a parental right [50]. Although an opt-out consent strategy was supported by our data, whether this would be accepted in Iquitos needs to be further studied, especially since distrust in health providers was identified as an important barrier to vaccine acceptance amongst Peruvian parents [18]. Similarly, evidence suggests that having to sign a consent form generates parental distrust of the vaccine, as this is not required for other vaccinations [18,51]. If written consent is sought, research favors getting parents to sign the consent form at school enrolment [48], a moment that was viewed favorably by various participants. Such a strategy should therefore be explored within the Peruvian context.

Interestingly, although all participants viewed school-based vaccination as beneficial, a few also highlighted associated barriers, e.g., school absenteeism. This was associated with girls’ fear of the vaccine or familial reasons e.g., moving away from the city. It affects coverage rates and in the second case, makes completion of dose schedule complicated, due to loss of follow-up. Another barrier to school-based vaccination raised by a participant was teachers’ opposition to vaccines. Although this was an individual case, given the important role of teachers in the vaccination campaign, it is crucial to ensure they are supportive of the program, once again highlighting the importance of providing them with the appropriate information and training, A recent study looking at HPV vaccination programs conducted in low-income countries reported that a mixed model system, including school and health facility-based vaccination, achieved the best coverage [52]. Although some participants supported the use of house to house vaccination campaigns, it is associated with cold chain and logistical challenges [53] and the feasibility and economics of integrating this approach in remote areas of Peru is yet to be established. In addition, the region has a net primary school attendance rate of 91.7% [54]. Research suggests that attempting to reach out-of-school girls by running specific campaigns does not significantly improve coverage rates in countries with over 80% school enrolment [55]. Although not giving these girls an opportunity to get vaccinated might worsen health inequality, it suggests that the region should focus on improving the school-health center-based approaches.

Importantly, low vaccine acceptance in indigenous communities also needs to be addressed. Certain beliefs regarding the causes of cervical cancer present in the local population, for example, associating it with witchcraft, might make it difficult to shift social norms towards acceptance of the vaccination. Low coverage has been found in other studies looking at vaccination in ethnic minorities in South America [56]. In the Amazonian region of Peru, 51 indigenous groups exist, most of which speak their own language [57]. Distrust in western medicine and a strong belief for traditional medicine have shown to hinder vaccination uptake in such groups [56]. A systematic review looking at strategies to overcome vaccine hesitancy recommends focusing on multicomponent or dialogue-based interventions [58]. These includes addressing the community’s distrust by involving the community leaders in the program design, improving social mobilization, and using social and mass media to deliver information. They also highlight the need to tailor the strategies to the target population by considering the cultural context and the population’s reasons for hesitancy [58]. In the absence of access to free or low cost satellite internet for health care purposes, many remote areas do not have access to mass media and the internet and rely on information from either community members or health workers [59], it is therefore important to investigate what interventions might be most effective in such groups.

Finally, although participants were supportive of the program, some felt their opinion was not considered by policy makers. Given that they have the most hand-on experience, they can provide a valuable insight into the health needs of the population and can make an important contribution to shaping and strengthening effective policy [60,61] and should therefore be included in the decision making process.

Overall, this study’s findings suggest that future strategies should be directed towards building knowledge amongst professionals and raising awareness within the general population, especially with the parents of girls eligible for the vaccination. Such campaigns should focus on reducing fear of side effects. New campaigns should potentially target less accessible areas and especially indigenous groups where cultural and social norms, affecting health beliefs and attitudes, may make increasing vaccination coverage difficult.

### Strengths and limitations

As far as the authors are aware, this qualitative study is the first to explore professionals’ perceived barriers and facilitators to the uptake of the HPV vaccination in Peru. A1, who is a native Spanish speaker, conducted the interviews in the local language. This facilitated richer narratives and avoided the need to use interpreters, which can be associated with problems related to misinterpretation of meaning or significance of participants’ responses [62].

Furthermore, the use of a theoretical framework, the SEM, allowed the study to examine the importance of numerous factors, at different levels, on vaccine uptake. Given that most vaccination uptake studies focus on interpersonal level variables [63], using such a model provides a useful holistic overview of the problem. This is particularly relevant given that research suggests that public health interventions to improve vaccination uptake targeting multiple levels of the model might be more effective [64].

It is important to be cautious when applying the conclusions beyond the sample and to evaluate whether they are transferable to other settings. Transferability can be defined as the degree to which qualitative studies’ findings can be transferred to other contexts or settings [65]. The use of purposive sampling to obtain a diverse range of opinions and experiences increase this study’s findings’ transferability [65] and so the findings are likely to be transferable to similar populations and settings. The adequacy of the final sample size was carefully monitored during the research process to ensure that the overall sample and associated data had sufficient information power to develop new knowledge in relation to the research questions [66]. Further to this, we had multiple analysts to support investigator triangulation and ensure that the data were viewed and interpreted via multiple lenses [67].

The impact of the main researcher also needs to be reflected upon. A1 had a central role in both data collection and analysis. Researcher bias was minimized by having a second coder review a sample of the transcripts and the wider research team supporting analysis and interpretation. To minimize the risk of A1’s (Spanish, female medical student) preconceptions and cultural values affecting data collection and interpretation, constant communication and support from the local supervisor and wider research team was sought.

## Conclusions

The current study contributes to the understanding of barriers and facilitators to the HPV vaccination program in Iquitos, Peru. Our findings highlight the importance of raising awareness among the general population and empowering communities to facilitate vaccination uptake. Future strategies should also be directed towards building more in-depth knowledge and improving face-to-face communication amongst professionals. Furthermore, policy makers should work closely alongside clinicians and teachers to ensure their experience and views are considered. In addition, a revision of the current consent procedure should be considered. Future research is needed to identify the most effective strategies to deliver information and to reduce existing knowledge deficits and address concerns.

## Supporting information

S1 File Interview topic guide. (DOCX)

## References

[1] Crosbie EJ, Einstein MH, Franceschi S, Kitchener HC. Human papillomavirus and cervical cancer. Lancet. 2013;382(9895):889–99. doi: 10.1016/S0140-6736(13)60022-7 23618600

[2] Schiffman M, Castle PE, Jeronimo J, Rodriguez AC, Wacholder S. Human papillomavirus and cervical cancer. Lancet. 2007;370(9590):890–907. doi: 10.1016/S0140-6736(07)61416-0 17826171

[3] Sung H, Ferlay J, Siegel RL, Laversanne M, Soerjomataram I, Jemal A, et al. Global Cancer Statistics 2020: GLOBOCAN Estimates of Incidence and Mortality Worldwide for 36 Cancers in 185 Countries. CA Cancer J Clin. 2021;71(3):209–49. doi: 10.3322/caac.21660 33538338

[4] Bosch FX. Eradication of cervical cancer in Latin America. Salud Publica Mex. 2016;58(2):97–100. 27557367

[5] Sargent A, Bailey A, Almonte M, Turner A, Thomson C, Peto J, et al. Prevalence of type-specific HPV infection by age and grade of cervical cytology: data from the ARTISTIC trial. Br J Cancer. 2008;98(10):1704–9. doi: 10.1038/sj.bjc.6604324 18392052 PMC2391119

[6] PATH, Nutrition Research Institute. Shaping a Strategy to Introduce HPV Vaccines in Peru: Formative Research Results from the HPV Vaccines: Evidence for Impact Project. Seattle PATH; 2009 [cited 2019 20 March]. Available from: https://www.who.int/immunization/hpv/target/shaping_strategy_introduce_hpv_vaccines_peru_path_2009.pdf.

[7] Kaiser J. Price Is the Main Barrier to Wider Use of Papillomavirus Vaccine. Science. 2008;320(5878):860. doi: 10.1126/science.320.5878.860 18487164

[8] Bruni L, Albero G, Serrano B, Mena M, Gómez D, Muñoz J, et al. Human Papillomavirus and Related Diseases in Peru. Summary Report 17 June 2019. [cited 2019 March 20]. Available from: https://hpvcentre.net/statistics/reports/PER.pdf.

[9] Ministerio de Salud del Perú. Resolución Ministerial 719-2018/MINSA. 2018 [cited 2019 22 March]. Available from: https://www.gob.pe/institucion/minsa/normas-legales/178240-719-2018-minsa.

[10] Ministerio de Salud del Perú. Directiva Sanitaria N°O64-MINSA/DGSP.V.01 Directiva Sanitaria para la Administración de la Vacuna Contra el Virus del Papiloma Humano (VPH) 2015 [cited 2019 March 22]. Available from: http://www.saludarequipa.gob.pe/redislay/descargas/Preliminar_Directiva_Vacuna_VPH.pdf.

[11] Voces Ciudadanas. Cobertura histórica vacunación contra el VPH según región de salud Perú 2011–2015. 2016 [cited 2019 March 20]. Available from: https://vocesciudadanas.pe/imagenes/COBERTURA%20HISTO%cc%81RICA%20VACUNACIO%cc%81N%20CONTRA%20EL%20VPH%202011-2016.pdf.

[12] Aguilar A, Pinto JA, Araujo J, Fajardo W, Bravo L, Pinillos L, et al. Control of cervical cancer in Peru: Current barriers and challenges for the future. Molecular and Clinical Oncology. 2016;5(2):241–5. doi: 10.3892/mco.2016.926 27446557 PMC4950606

[13] Ministerio de Salud del Perú. Guía técnica: Guía de practica clínica para la prevención y manejo del cáncer de cuello uterino. Tratado de Medicina. 2017:1–5.

[14] Almonte M, Albero G, Molano M, Carcamo C, García PJ, Pérez G. Risk factors for human papillomavirus exposure and co-factors for cervical cancer in Latin America and the Caribbean. Vaccine. 2008;26:L16–36. doi: 10.1016/j.vaccine.2008.06.008 18945400

[15] Instituto Nacional de Estadística e Informática. Encuesta Demográfica y de Salud Familiar—ENDES 2017 [cited 2019 March 22]. Available from: https://www.inei.gob.pe/media/MenuRecursivo/publicaciones_digitales/Est/Lib1525/index.html.

[16] Instituto Nacional de Estadística e Informática. Programa de prevención y control del cáncer 2016 [cited 2019 April 10]. Available from: https://www.inei.gob.pe/media/MenuRecursivo/publicaciones_digitales/Est/Lib1432/cap02.pdf.

[17] Lee FH, Paz-Soldan VA, Carcamo C, Garcia PJ. Knowledge and Attitudes of Adult Peruvian Women vis-à-vis Human Papillomavirus (HPV), Cervical Cancer, and the HPV Vaccine. Journal of Lower Genital Tract Disease. 2010;14(2):113–7. doi: 10.1097/LGT.0b013e3181c08f5e 20354419

[18] Bartolini RM, Winkler JL, Penny ME, LaMontagne DS. Parental Acceptance of HPV Vaccine in Peru: A Decision Framework. PLoS One. 2012;7(10):e48017. doi: 10.1371/journal.pone.0048017 23144719 PMC3483308

[19] Cates JR, Shafer A, Carpentier FD, Reiter PL, Brewer NT, McRee AL, et al. How Parents Hear about HPV Vaccine: Implications for Uptake. Journal of Adolescent Health. 2010;47(3):305–8. doi: 10.1016/j.jadohealth.2010.04.003 20708571 PMC2928162

[20] Guerry SL, De Rosa CJ, Markowitz LE, Walker S, Liddon N, Kerndt PR, et al. Human papillomavirus vaccine initiation among adolescent girls in high-risk communities. Vaccine. 2011;29(12):2235–41. doi: 10.1016/j.vaccine.2011.01.052 21288799

[21] Rosenthal SL, Weiss TW, Zimet GD, Ma L, Good MB, Vichnin MD. Predictors of HPV vaccine uptake among women aged 19–26: Importance of a physician’s recommendation. Vaccine. 2009;29(5):890–5. 10.1016/j.vaccine.2009.12.063 20056186

[22] Caskey R, Lindau ST, Alexander GC. Knowledge and Early Adoption of the HPV Vaccine Among Girls and Young Women: Results of a National Survey. Journal of Adolescent Health. 2009;45(5):453–62. doi: 10.1016/j.jadohealth.2009.04.021 19837351

[23] Niccolai LM, Hansen CE, Credle M, Shapiro ED. Parents’ Recall and Reflections on Experiences Related to HPV Vaccination for Their Children. Qualitative Health Research. 2016;26(6):842–50. doi: 10.1177/1049732315575712 25779984 PMC4573381

[24] Bahena M, Carvajal-Suarez M, Soliman AS, Luo J, De Alba A. The influence of medical providers on HPV vaccination among children of Mexican mothers: a comparison between Mexico and the Midwest region of the United States. BMC Public Health. 2019;19(1):515. doi: 10.1186/s12889-019-6718-0 31060527 PMC6501334

[25] Brewer NT, Fazekas KI. Predictors of HPV vaccine acceptability: A theory-informed, systematic review. Preventive Medicine. 2007;45(2):107–14. doi: 10.1016/j.ypmed.2007.05.013 17628649

[26] LaMontagne DS, Nghi NQ, Nga LT, Janmohamed A, Thanh Huyen DT, Hien NT, et al. Qualitative study of the feasibility of HPV vaccine delivery to young adolescent girls in Vietnam: evidence from a government-implemented demonstration program. BMC Public Health. 2014;14(556). doi: 10.1186/1471-2458-14-556 24898950 PMC4067078

[27] Instituto Nacional de Estadística e Informática. Perú: Estimaciones y Proyecciones de Población Total por Sexo de las Principales Ciudades, 2000–2015. Boletín Especial N 23 2012 [cited 2019 March 25]. Available from: http://proyectos.inei.gob.pe/web/biblioineipub/bancopub/Est/Lib1020/Libro.pdf.

[28] Instituto Nacional de Estadística e Informática. Estimaciones y Proyecciones de Población. 2015.

[29] Ministerio de Salud del Perú. Análisis de Situación de Salud de Loreto 2015 [cited 2019 March 22]. Available from: http://www.cdc.gob.pe/portal/Asis/indreg/asis_loreto.pdf.

[30] Instituto Nacional de Estadística e Informática. La Autoidentificación Étnica: Población Indígena y Afroperuana 2017 [cited 2019 April 12]. Available from: https://www.inei.gob.pe/media/MenuRecursivo/publicaciones_digitales/Est/Lib1642/.

[31] Jamshed S. Qualitative research method-interviewing and observation. Journal of basic and clinical pharmacy. 2014;5(4):87–8. doi: 10.4103/0976-0105.141942 25316987 PMC4194943

[32] Nyambe A, Van Hal G, Kampen JK. Screening and vaccination as determined by the Social Ecological Model and the Theory of Triadic Influence: a systematic review. BMC Public Health. 2016;16(1):1166. doi: 10.1186/s12889-016-3802-6 27855680 PMC5114823

[33] Glanz K, Rimer BK, Viswanath K. Health Behavior: Theory, Research, and Practice, 5th Edition. 5 ed: Jossey-Bass; 2015.

[34] Silverman D. Doing qualitative research. 2 ed. London: SAGE Publications; 2005.

[35] Etikan I. Comparision of Snowball Sampling and Sequential Sampling Technique. Biometrics & Biostatistics International Journal. 2016;3(1).

[36] Dicicco-Bloom B, Crabtree BF. The qualitative research interview. Med Educ. 2006;40(4):314–21. doi: 10.1111/j.1365-2929.2006.02418.x 16573666

[37] Hilton S, Hunt K, Bedford H, Petticrew M. School nurses’ experiences of delivering the UK HPV vaccination programme in its first year. BMC Infectious Diseases. 2011;11(1):226. doi: 10.1186/1471-2334-11-226 21864404 PMC3176210

[38] Cartmell KB, Young-Pierce J, McGue S, Alberg AJ, Luque JS, Zubizarreta M, et al. Barriers, facilitators, and potential strategies for increasing HPV vaccination: A statewide assessment to inform action. Papillomavirus Research. 2018;5:21–31. doi: 10.1016/j.pvr.2017.11.003 29248818 PMC5886972

[39] Braun V, Clarke V. Using thematic analysis in psychology. Qualitative Research in Psychology. 2006;3(2):77–101.

[40] Kolb SM. Grounded theory and the constant comparative method: valid research strategies for educators. Journal of Emerging Trends in Educational Research and Policy Studies. 2012;3(1):83–6.

[41] Instituto Nacional de Estadística e Informática. III Censo de Comunidades Nativas 2017 Resultados Definitivos 2018 [cited 2019 April 12]. Available from: https://www.inei.gob.pe/media/MenuRecursivo/publicaciones_digitales/Est/Lib1598/TOMO_01.pdf.

[42] Ishiwada N, Suzuki C, Hasebe S, Tsuchiya A, Takeuchi N, Hishiki H, et al. The effects of health education on health science teachers’ intention to recommend adolescent HPV vaccine for female students in Japan. Hum Vaccin Immunother. 2020;16(11):2752–7. doi: 10.1080/21645515.2020.1732163 32159443 PMC7734136

[43] Vorsters A, Bosch FX, Bonanni P, Franco EL, Baay M, Simas C, et al. Prevention and control of HPV infection and HPV-related cancers in Colombia- a meeting report. BMC Proc. 2020;14:8. doi: 10.1186/s12919-020-00192-2 32577128 PMC7307134

[44] Mendes Lobão W, Duarte FG, Burns JD, de Souza Teles Santos CA, Chagas de Almeida MC, Reingold A, et al. Low coverage of HPV vaccination in the national immunization programme in Brazil: Parental vaccine refusal or barriers in health-service based vaccine delivery? PLoS One. 2018;13(11):e0206726. doi: 10.1371/journal.pone.0206726 30418980 PMC6231618

[45] Farias CC, Jesus DV, Moraes HS, Buttenbender IF, Martins IS, Souto MG, et al. Factors related to non-compliance to HPV vaccination in Roraima-Brazil: a region with a high incidence of cervical cancer. BMC Health Serv Res. 2016;16(1):417. doi: 10.1186/s12913-016-1677-y 27550325 PMC4994290

[46] Podolsky R, Cremer M, Atrio J, Hochman T, Arslan AA. HPV Vaccine Acceptability by Latino Parents: A Comparison of U.S. and Salvadoran Populations. Journal of Pediatric and Adolescent Gynecology 2009;22:205–15. doi: 10.1016/j.jpag.2008.05.010 19646665

[47] Instituto Nacional de Estadística e Informática. Perú: Indicadores de Educación por Departamentos 2009–2019 2020 [cited 2020 15 November]. Available from: https://www.inei.gob.pe/media/MenuRecursivo/publicaciones_digitales/Est/Lib1751/libro.pdf.

[48] Kabakama S, Gallagher KE, Howard N, Mounier-Jack S, Burchett HE, Griffiths UK, et al. Social mobilisation, consent and acceptability: a review of human papillomavirus vaccination procedures in low and middle-income countries. BMC Public Health. 2016;16(1):834. doi: 10.1186/s12889-016-3517-8 27543037 PMC4992325

[49] World Health Organization. Considerations regarding consent in vaccinating children and adolescents between 6 and 17 year old (WHO/IVB/14.04): World Health Organization; 2014 [cited 2019 April 12]. Available from: https://www.who.int/immunization/programmes_systems/policies_strategies/consent_note/en/.

[50] Pitts MJ, Tufts KA. Implications of the Virginia Human Papillomavirus Vaccine Mandate for Parental Vaccine Acceptance. Qualitative Health Research. 2012;23(5):605–17. doi: 10.1177/1049732312470871 23275459

[51] Howard N, Gallagher KE, Mounier-Jack S, Burchett HED, Kabakama S, LaMontagne DS, et al. What works for human papillomavirus vaccine introduction in low and middle-income countries? Papillomavirus Res. 2017;4:22–5. doi: 10.1016/j.pvr.2017.06.003 29179865 PMC5710981

[52] Ladner J, Besson MH, Hampshire R, Tapert L, Chirenje M, Saba J. Assessment of eight HPV vaccination programs implemented in lowest income countries. BMC Public Health. 2012;12:370. doi: 10.1186/1471-2458-12-370 22621342 PMC3419135

[53] World Health Organization. Guide to Introducing HPV Vaccine into National Immunization Programmes 2016 [cited 2019 April 14]. Available from: https://www.who.int/immunization/diseases/hpv/HPV_vaccine_intro_guide_draft_Oct2016.pdf.

[54] Ministerio de Educación del Perú. Loreto: ¿cómo vamos en educación? 2017 [cited 2019 March 19]. Available from: http://escale.minedu.gob.pe/documents/10156/4228634/Perfil+Loreto.pdf.

[55] Gallagher KE, Howard N, Kabakama S, Mounier-Jack S, Griffiths UK, Feletto M, et al. Lessons learnt from human papillomavirus (HPV) vaccination in 45 low- and middle-income countries. PLoS One. 2017;12(6):e0177773. doi: 10.1371/journal.pone.0177773 28575074 PMC5456063

[56] Burghouts J, Del Nogal B, Uriepero A, Hermans PW, de Waard JH, Verhagen LM. Childhood Vaccine Acceptance and Refusal among Warao Amerindian Caregivers in Venezuela; A Qualitative Approach. PLoS One. 2017;12(1):e0170227. doi: 10.1371/journal.pone.0170227 28107501 PMC5249092

[57] Ministerio de Cultura del Perú. Lista de Pueblos Indígenas u Originarios 2018 [cited 2019 March 19]. Available from: https://bdpi.cultura.gob.pe/pueblos-indigenas.

[58] Jarrett C, Wilson R, O’Leary M, Eckersberger E, Larson HJ, SAGE Working Group on Vaccine Hesitancy. Strategies for addressing vaccine hesitancy—A systematic review. Vaccine. 2015;33(34):4180–90. doi: 10.1016/j.vaccine.2015.04.040 25896377

[59] Ministerio de Transportes y Comunicaciones del Perú. Problemática de las comunicaciones rurales 2013 [cited 2019 April 10]. Available from: http://www.midis.gob.pe/dmdocuments/fonie_taller_02problematica_telecom_rurales_fitel.pdf.

[60] Aroskar MA, Moldow DG, Good CM. Nurses’ voices: Policy, practice and ethics. Nursing Ethics. 2004;11(3):266–76. doi: 10.1191/0969733004ne694oa 15176640

[61] Shariff N. Factors that act as facilitators and barriers to nurse leaders’ participation in health policy development BMC Nursing 2014;13(20). doi: 10.1186/1472-6955-13-20 25053921 PMC4105513

[62] Squires A. Methodological challenges in cross-language qualitative research: a research review. Int J Nurs Stud. 2009;46(2):277–87. doi: 10.1016/j.ijnurstu.2008.08.006 18789799 PMC2784094

[63] Boerner F, Keelan J, Winton L, Jardine C, Driedger SM. Understanding the interplay of factors informing vaccination behavior in three Canadian provinces. Hum Vaccin Immunother. 2013;9(7):1477–84. doi: 10.4161/hv.24427 23571169

[64] Kumar S, Quinn SC, Kim KH, Musa D, Hilyard KM, Freimuth VS. The social ecological model as a framework for determinants of 2009 H1N1 influenza vaccine uptake in the United States. Health Educ Behav. 2012;39(2):229–43. doi: 10.1177/1090198111415105 21984692 PMC3916095

[65] Anney VN. Ensuring the Quality of the Findings of Qualitative Research: Looking at Trustworthiness Criteria. Journal of Emerging Trends in Educational Research and Policy Studies. 2014;5:272–81.

[66] Malterud K, Siersma VD, Guassora AD. Sample Size in Qualitative Interview Studies: Guided by Information Power Qualitative Health Research. 2016;26(13):1753–60. doi: 10.1177/1049732315617444 26613970

[67] Carter N, Bryant-Lukosius D, DiCenso A, Blythe J, Neville AJ. The Use of Triangulation in Qualitative Research Oncology Nursing Forum 2014;41(5):545–7 doi: 10.1188/14.ONF.545-547 25158659

