# Supplementary material for: Nurses’ and teachers’ perceived barriers and facilitators to the uptake of the Human Papilloma Virus (HPV) vaccination program in Iquitos, Peru: A qualitative study
Source: PLoS One. 2021 Jul 29;16(7):e0255218. doi: 10.1371/journal.pone.0255218 (PMC8320989; doi:10.1371/journal.pone.0255218)
Supplement: S1 File — (DOCX) [file pone.0255218.s001.docx]

**S1 File: Interview Topic Guide**

**Introduction:**

Hello, my name is Anna and I am a medical student at the University of Birmingham medical school. I will be interviewing you today. Before we start, I am just going to give you a brief explanation about the study and clarify any questions you may have.

**Purpose:** This study aims to explore the facilitators and barriers to the implementation of the HPV vaccination program by interviewing nurses and teachers. We hope to use the results to help inform future strategies to improve the coverage rates.

**Data Collection:**

- With your permission, the interview will be audio-recorded using this encrypted, password-protected, digital recording device.
- Everything that is said during the interview will be transcribed so that the information can be analyzed.
- Everything you say in this interview is strictly confidential.
- The whole interview is expected to take 45-60 minutes
- We will use anonymous quotations from the interviews in our research article. No identifiable data will be published, so you will not be identified in any way.

**Questions:** Before we proceed, do you have any questions?

**Consent:**

- You have provided written consent, but can you confirm that you understand everything I have said and that you agree for the interview to take place?
- Can you also confirm that you understand that you can withdraw from the study without giving a reason?
- If you need to stop the interview at any point, then please let me know.

| **Topic** | **Questions** *(& probes)* |
| --- | --- |
| Personal | Could you please tell me a bit about your work? Your role within the HPV vaccination program? |
| Knowledge about HPV and vaccination | What do you know about HPV?  What do you know about the vaccine? *How many cancers can it prevent? Effectiveness?*  Where do you get your information about HPV/the vaccine?  Have you ever attended training specific on vaccine delivery?  What information would you like to have?  Do you run educational events for HPV?  How safe do you think the vaccination is? |
| Perceptions about barriers and facilitators to HPV vaccination | What are your thoughts about the HPV vaccination in the region?  What factors in your region facilitate HPV vaccination?  What are barriers to HPV vaccination in your region?  *How can they be addressed?* |
| Recommendation | What strategies are you aware of that have been used to improve vaccination rates?  *How well did they work? How could these strategies be improved?*  What type of strategies do you think would be most effective in the region?  Policy/legislation as a means to increase vaccination can be vary varied e.g. awareness campaigns, mandating vaccination for school attendance.  *What are your preferences about the level of policy/legislation to increase HPV vaccination in the region?*  Thinking about policy/legislation, what strategy would you suggest to increase vaccination rates?  Tell me about any collaboration with national stakeholders that you have been involved with to improve vaccination?  *Was it effective?*  Who do you think are the key stakeholders who should be included in developing strategies to maximize HPV vaccination rate? |
| School system | Can you tell me about any HPV education that is include as part of the school curriculum?  *Do you also discuss other issues related to sexuality?*  How do you feel that schools could increase the rate of vaccination?  What do you think are advantages/disadvantages of providing vaccinations in school? |
| Closing question | What else about HPV vaccination is important for us to know?  *Would you like to add anything?* |

**Thank you for taking part.** These were all the questions we wanted to ask you. Do you have any questions? Please don’t hesitate to contact me. The contact details are in the participant information leaflet.
